# Coping among trauma-affected youth: a qualitative study

**DOI:** 10.1186/s13031-015-0062-5

**Published:** 2015-11-17

**Authors:** Megan Cherewick, Anjalee Kohli, Mitima Mpanano Remy, Clovis Mitima Murhula, Arsene Kajabika Bin Kurhorhwa, Alfred Bacikenge Mirindi, Nadine Mwinja Bufole, Jean Heri Banywesize, Gisele Mushengezi Ntakwinja, Gracia Mitima Kindja, Nancy Glass

**Affiliations:** Department of International Health, Johns Hopkins Bloomberg School of Public Health, 615 N. Wolfe Street, 21204 Baltimore, MD USA; Johns Hopkins School of Nursing, Baltimore, MD USA; Programme d’Appui aux Initiatives Economiques (PAIDEK) Pigs for Peace, Bukavu, Democratic Republic of Congo; Pigs for Peace, Bukavu, Democratic Republic of Congo

**Keywords:** Coping, Conflict-affected, Youth, War, Trauma, Adolescent, Resilience

## Abstract

**Background:**

Eastern Democratic Republic of Congo has endured decades of conflict resulting in widespread experiences of conflict related trauma and destruction to health and social infrastructure. The aim of this qualitative study was to provide a context specific understanding of youth exposure to violence (ages 10–15 years) and use of cognitive and behavioral coping strategies.

**Methods:**

A purposive sampling strategy based on age, gender and exposure to traumatic events was used to identify eligible youth in an ongoing parent study from four villages in the Walungu Territory, Eastern Democratic Republic of Congo. These four villages were selected from a total of 10 participating in the parent study because of the reported high exposure to conflict-related trauma. The interview guide consisted of broad open-ended questions related to the following topics, 1) identification of traumatic experiences, 2) methods for coping and changes in coping behavior 3) gender and age differences in coping, 4) sources of psychosocial support. A grounded theory approach was used to identify emergent themes.

**Results:**

Of the 48 eligible participants identified, 30 youth completed the interview, 53 % were female (*n* = 16) and 47 % were male (*n* = 14). Youth ranged in age from 10–15 (mean age = 13.07). Exposures to different forms of violence and stress were reported among youth participants. Exposures to traumatic stressors occur at the individual, family and community level. In response to traumatic stress, youth reported both cognitive and behavioral coping strategies. Cognitive coping strategies included trying to forget and praying. Behavioral coping strategies included social support seeking and risk-taking behavior. These strategies may be used in mutually reinforcing ways, with youth employing more than one coping strategy.

**Conclusion:**

This qualitative research provides important, culturally grounded information on coping strategies used by youth in rural post-conflict settings where limited psychosocial support services are available. Understanding use of cognitive and behaviors coping strategies may inform local community and international development programs to support youth mental health along adaptive trajectories resulting in promotion of well-being and reduced risk taking behaviors.

## Background

### Democratic Republic of Congo: rebuilding after prolonged conflict

The Democratic Republic of Congo (DRC) has endured decades of destruction to health and social infrastructure. A history of colonialism, theft of the DRC’s enormous mineral wealth and strategic ‘pitting’ of ethnic groups against one another set the stage for prolonged conflict including two conflicts with neighboring countries. Within this context, families are striving to rebuild their households and communities and improve the life and livelihoods of future generations.

A cross-sectional study in the Ituri province in the eastern DRC investigated conflict-related traumatic events and found that among 477 girls and 569 boys ages 13–21, 95 % of the respondents reported at least one traumatic event; on average, adolescents were exposed to 4.7 traumatic events over their lifetime, and 52 % of adolescents met the criteria for PTSD [1]. Violence, population displacement and the destruction of health and educational institutions have weakened the local systems required to respond to children’s mental health and promote well-being.

### Armed conflict and mental health of children

Worldwide, UNICEF estimates that over one billion children under the age of eighteen are affected by conflict (UNICEF, 2009). Globally, an estimated two million children have lost their lives over the past decade to armed conflict, six-million have been left severely injured or disabled, twelve million have been left destitute and 300,000 children have served as child soldiers [2]. The stress and trauma of armed conflict is devastating to psychological well-being and healthy development of children. The developmental period between childhood and adolescence is a particularly vulnerable period of time characterized by, “complex and multiple changes across biological, psychological and interpersonal domains” [3]. The psychological impact of armed conflict on children includes increased prevalence of PTSD, anxiety, depression [4], psychophysiological disturbances such as nightmares and trouble sleeping, fear, grief, behavioral problems [5], and changes in educational attainment and performance, lack of hope and personality changes [6]. Researchers report that children are aware of stress and their coping strategies due to psychological trauma and are able to identify stressors, describe coping strategies and evaluate the effectiveness of those strategies at ages as young as six years old [7].

To date, most research on conflict affected children has focused on PTSD [8] with less research focused on depression and behavioral problems. Pooled prevalence estimates from 17 armed conflict affected countries found that the impact of armed conflict resulted in 47 % of children with moderate to severe diagnosis of PTSD, 43 % with depression and 27 % with anxiety [2]. Armed conflict can result in psychological distress in the short term, but can also lead to long-term psychopathology. Research with former child soldiers in Mozambique found 50 % of participants reported traumatic stress reactions sixteen years after return to civilian life [9]. Furthermore, protracted conflict involving non-state actors such as rebel groups can directly harm child development when children witness or experience conflict-related violence and can also indirectly harm children’s well being by weakening health care delivery systems, disrupting schools and destroying infrastructure [10].

However research has also shown that youth exposed to armed conflict do not necessarily develop psychopathology and in contrast, many youth demonstrate remarkable resilience. Norman Garmezy is often credited with developing the field of resilience research with pioneering research conducted in the early 1970s focusing on positive adaptation for children at risk for psychopathology [11, 12]. Researchers since have began to focus on resilience, seeking to identify factors that allowed some individuals to cope better than others when faced with adversity [12–16]. Some of the factors that protect against psychological distress and promote psychological well-being at the individual level include female gender, higher intellectual capacity, political activity, problem-solving, seeking social support and demonstrating faith in a higher power or religion [14, 17, 18]. Research has also identified factors related to an individual’s external social ecology including family and community level factors. Protective factors at the family level include family support and stability and parental monitoring [19–21]. At the community level, attachment to community and peers through school or churches, community acceptance and access to social support were associated with youth resilience [19, 22–24]. While research has identified the importance of investigating factors related to positive adaptation, less is known about how youth’s individual coping strategies are implemented within specific cultural contexts. More research is needed to better understand which coping strategies youth employ in the Democratic Republic of Congo and how these strategies have the potential to help or harm mental health in conflict-affected youth.

### Theoretical background on coping

Lazarus and Folkman (1984) originated the term “coping” to describe responses to stress [25]. Lazarus and Folkman defined stress as a condition or feeling experienced when a person appraises an event as “exceeding his or her resources and as endangering well-being” [25]. This theory posited that the best way to measure coping was through an individual’s personal appraisal. Personal appraisal refers to the various ways individuals seek to modify adverse aspects of their life to minimize the internal threat of stressors. Appraisal can be primary (perception of a stressor) or secondary (evaluation of potential effectiveness and consequences of coping behaviors) [25]. Coping is effective if stress is accurately appraised and specific behavioral and cognitive strategies are used to manage, reduce or tolerate stressful events [26].

The cognitive and behavioral coping strategies used by youth have been grouped in different ways. Lazarus and Folkman distinguish coping styles as emotion centered coping which seeks to regulate internal emotions and the meaning of an event and problem-based coping which aims to change the problem or conflict [25]. Other researchers have grouped coping in different ways. Beehr and McGrath distinguish more categories of coping styles and group styles as preventive, anticipatory, dynamic, reactive and residual coping [27]. Ghimbulut categorized coping styles as, “emotion coping” (focusing on changing one’s own emotions), behavioral coping (directed toward the cause of the problem) and cognitive coping (directed on one’s own beliefs) [28]. A study with war-affected children in Croatia found six distinct coping strategies including aggressive activities, problem oriented, avoidance and relaxation, emotion expression and social support seeking [6].

While some research suggests that emotion oriented coping behaviors are associated with poorer mental health and problem based coping behaviors are associated with better mental health outcomes [29, 30], other researchers have questioned whether emotion focused coping should be considered maladaptive. For example, in environments such as humanitarian emergencies and armed conflict, engagement (problem focused) coping may be a less appropriate coping method than disengagement (emotion focused) coping because youth may be powerless to actively change stressors related to the conflict/disaster and instead emotion focused coping may be a positive strategy, more easily accessible than problem focused strategies.

Considering the cultural context in which coping strategies are employed is essential to gain depth of meaning to motivations for employing a particular strategy and the positive or negative benefits of using a particular strategy. Qualitative case-study research with Cambodian refugees found that avoidant coping was utilized with traumatized Cambodian refugees who sought to avoid thoughts, behaviors and activities that reminded them of the past and linked this coping strategy to a history of “dishonorable events in Cambodian history” and collective shame felt by Cambodians [31]. The Cambodian belief system perceives personal bad fortunes stem from dishonorable events in a previous life and therefore led individuals to use avoidant coping strategies rather than more problem focused strategies. Qualitative narrative research with 14 Sudanese youth refugees found that a sense of communal self was thematic in interviews and that suppression and distraction were a common coping strategy [32]. Participants used distraction to avoid difficult thoughts and feelings and believed this strategy helped “protect themselves from feelings that they feel powerless to handle” [32]. Research with Zimbabwean adolescents found that there was greater use of emotion focused strategies rather than problem solving strategies because cultural norms in Zimbabwe discourage problem solving strategies that may involve confrontation or challenging elders and instead youth favor distancing, keeping to themselves and other emotion focused strategies that may be more characteristic of a collectivistic society versus an individualistic society [33]. Whereas in Western societies coping strategies are often connected to an individualistic approach whereby individuals seek help through youth counseling, in developing countries and contexts where youth have experienced armed conflict, the coping approach may be more collective in nature, with greater use of community support systems to support emotion focused coping strategies rather than problem focused strategies. In addition, cultural norms can shape use of coping strategies. For example, a mixed method study among Palestinian refugee youth (ages 8–17) living in Gaza found that girls were less likely to use coping strategies that would require them to be far from home and used more strategies that involved being close to home such as praying in the home, whereas boys were more likely to use leisure activities and relaxation activities outside of the home [34]. Cultural gender role expectations can influence types of coping strategies utilized by youth.

In the Democratic Republic of Congo, recent research questions whether disengagement (emotion focused) coping should be construed as a maladaptive reaction to conflict affected situations, and argues a more detailed, context specific understanding is needed to understand youth coping strategies [35]. This research in the DRC, among 952 armed conflict affected youth ages 13–21, found that in response to traumatic exposure, disengagement coping behaviors, such as distraction, resignation and social withdrawal were more common among adolescents as compared to engagement behaviors such as cognitive restructuring or problem solving [35]. Qualitative research is needed from a youth perspective to better define and gain depth of knowledge as to the cognitive and behavioral coping strategies. As noted above, research has stressed the importance of context in understanding variations in use of coping strategies. Documenting how conflict affected children cope with adversity will improve our understanding of the types of cognitive and behavioral strategies utilized by youth and relationships with individual, family and community factors, thus informing youth-based programs in conflict and post-conflict settings.

### Research aim

The purpose of the qualitative study is to examine youth (ages 10–15 years) exposure to violence and other traumatic events, use of cognitive and behavioral coping strategies and the (contingent) relationships of these coping strategies with family and community. The principal objectives of study are to document youth defined coping strategies, to consider how coping may inform adaptation trajectories over time and to consider opportunities to support coping strategies that promote well-being and better mental health outcomes. This study will provide a context specific understanding of mental health and the role of coping strategies following exposure to diverse and multiple traumatic events. This qualitative work will also be used to adapt existing coping scales for youth for use in future quantitative phases of this research. By providing a culturally grounded basis for developing a comprehensive framework of coping, this research will provide the basis for empirical testing of the relationship between use of coping strategies and mental health and well-being. The findings can be used to broaden the discourse on coping strategies utilized by conflict affected youth and to identify factors and relationships that support healthy coping strategies.

### Methods

#### Study design and setting

This study uses the research infrastructure of a larger National Institute of Health (NIH)/National Institute of Child Health and Human Development (NICHD) funded randomized community trial of a youth-led livestock microfinance program, Rabbits for Resilience (RFR). RFR is a collaborative project between Programme d’Appui aux Inititatives Economiques (PAIDEK), an established Congolese microfinance institute, and Johns Hopkins University School of Nursing (JHUSON). RFR is designed to increase youth and family resilience to ultimately improve health and emotional adjustment after exposure to adversity and trauma. RFR includes male and female youth ages 10–15 years living in 10 rural villages in the Walungu territory in Eastern Democratic Republic of Congo. The ten villages included in the impact evaluation of RFR were selected for several reasons including; (1) feasibility of delivering an intervention over a wide geographical area; (2) commitment to the intervention and study by traditional chiefs and administrators; and (3) findings from village-level assessments that showed few health and development programs exist in the area, including microfinance. The people living in these rural villages have experienced significant violence, displacement and trauma over the past 20 years with limited health care, schools or governmental or non-governmental organizations to provide support and resources.

#### Theoretical perspective

In order to understand youth participant exposure to violence and other traumatic events and their cognitive and behavioral coping strategies, a grounded theory approach was used in the initial coding of the data and deductive reasoning used to explore how emergent themes relate to existing theory [36]. The grounded theory methodological approach is grounded in the constructivist epistemology, that meaning is co-created in the discourse between people. The grounded theory approach supports the role of subjectivity in creation of meaning. Moreover, gaining knowledge through discourse is necessarily grounded in a particular social and historical *context*. For this study a grounded theory perspective is valuable because of the implicit focus on taking Congolese youth comments as true representations of their perspective on trauma and coping. Second, a deductive process was used whereby emergent themes were categorized based on existing theory suggesting two coping domains, cognitive and behavioral coping strategies. This study utilizes in depth interviews and relies on the dialogue between interviewer and interviewee to construct an improved understanding of ways youth cope with stress.

#### Sample

A purposive sampling strategy was used to identify eligible youth enrolled in the parent study. Investigating types of exposure to violence and other traumatic events that youth experienced was important to understand contextual variables that can affect coping and resulting mental health outcomes. At baseline, youth completed a survey including the Harvard Trauma Questionnaire. Youth were selected from four of the 10-study villages. These four villages were selected because of the reported high exposure to conflict-related trauma. Within these villages, baseline data from the parent study was used for purposive sampling based on age, gender and exposure to traumatic events to identify 48 youth (12 from each village). Traumatic exposures were represented by a wide range of experiences including murder of family/friends, having ill health without care, lacking food and water, being seriously injured, being close to death, separation from family, experiences in combat and brainwashing. Specifically, youth were selected for variation on experience of trauma: low exposure to trauma (0–1 events), medium exposure (2–3 events) and high exposure (4 or more events). Within each trauma exposure level purposive sampling involved achieving a balanced distribution of participants by age [11–16] and gender. Of the 48 eligible participants identified, 30 youth completed the interview (16 were not available on the days of fieldwork).

#### Congolese research team and development of the youth interview guide

Congolese research team members were previously trained by the parent study team and had successfully completed qualitative and quantitative research in the study villages. Congolese interviewers actively participated in the design, development, piloting and revision of the youth interview guide. The research team reviewed and revised interview questions to ensure questions were culturally relevant and would be appropriate for ages 10–15. The questionnaire was translated by the Congolese research team into local languages, Swahili and Mashi, used for interview with youth. Probes were developed with local partners to capture greater depth in participant responses. The interview guide was piloted in June 2014 among 5 youth in the microfinance demonstration project site located in a village outside of Bukavu, the capital city of South Kivu province. Following the pilot test, the research team revised the interview guide and conducted a second pilot test among 5 different youth. Revisions included the removal of redundant questions to shorten length, rewording of questions found to be confusing or unclear and minor re-ordering of questions to promote improved flow of the instrument as a whole. The final guide consisted of broad open-ended questions related to the following topics, 1) identification of trauma-related experiences, 2) methods for coping and changes in coping behavior 3) respondent’s perception of gender and age differences in coping, 4) sources of psychosocial support. After final revisions to the interview guide, the researcher commenced two day team training in administration of consent (in alignment with IRB regulations), ethics, and qualitative interview methods.

#### Procedures

The Johns Hopkins School of Medicine Institutional Review Board (IRB) approved this study (IRB: CIR00001977; Date: 06-23-14). The research team also received approval to conduct the research with local partners PAIDEK by village traditional and administrative leaders. Parents/caregivers of eligible youth were provided with the purpose of the study, risks and benefits of participation in the study and then were asked to provide verbal informed consent for their child to participate. If a parent/caregiver consents, their child was then asked for verbal assent after receiving details on the purpose of the study and prior to beginning the interview. No participants’ names were recorded, all interviews were conducted in private and no information was shared outside the research team.

After parents/caregivers provided informed consent and youth provided assent, the interviewer selected a location within the village for the interview, the location was away from parents/caregivers and friends that would allow for privacy and disclosure during the interview. The interviewers started with asking youth participants to describe their typical day, their family, their community and activities they participate in. Initial questions allowed the interviewer to develop a level of rapport with the youth where they would feel comfortable answering more personal questions. The interviewer utilized probes to explore topics related to coping strategies, trauma and family and community relationships in greater depth. The final interview guide resulted in interviews between 30 and 60 min in length. All participant answers were recorded verbatim and participants were provided with compensation for their time equal to 2USD, an amount considered appropriate after consultation with village leaders and research team members.

### Analysis

After completion of interviews, a Congolese translator completed translation of all transcripts from French or the local language (Swahili or Mashi) to English. The translations were cross-checked by researchers based in the US and in consultation with the Congolese research team. After completion of the translation and review, the analysis used a grounded theory approach, which is rooted in a participatory transformative paradigm [36, 37]. First initial codes were developed through line-by-line coding of a sub-sample of interviews (interviews of 5 girls and 5 boys). *Line-by-line* coding involves providing a code to each line of written data and allows for ideas to emerge that may have escaped attention if reading for a general thematic analysis [36]. Line-by-line coding allows the researcher to identify implicit actions and meanings, identify gaps in the data and to note common relationships and significance between codes. Where appropriate, codes were left in vivo, to preserve participants’ language and meaning. In vivo codes are locally defined terms that condense meaning and are characteristic of societies and reflect assumptions, actions and imperatives [36]. The next analysis phase was the development of *focused codes* that were applied to all interviews. Focused codes are developed from using the most significant and/or frequent initial codes to make analytic sense of the data. Next, *axial coding* was used to represent the content of focused codes and to relate common codes, categories and concepts to each other. Second, a deductive process was used whereby emergent themes were categorized based on application of existing theory suggesting two coping domains, cognitive and behavioral coping strategies. Final coding structure was applied to each transcript using Atlas Ti software.

During the application of focused and axial coding, memos were written by the researchers to help identify emergent themes related to youth coping styles. Memos allow the researcher to capture comparisons and connections and to construct analytic notes [36]. Iterative inductive content analysis was used to identify emergent themes and connections between themes [38]. Verbatim statements that capture emergent themes were identified for use as quotes.

## Results

Sample demographics are shown in Table 1. Of the 48 eligible participants identified, 30 youth completed the interview, 53 % were female (*n* = 16) and (47 %) were male (*n* = 14). Youth ranged in age from 10–15 years old (mean age = 13.07) and represented 4 villages.

**Table 1:** Sample Demographics: Trauma Exposure, Gender and Age

	Girls		Boys		
Trauma Exposure	Ages 10–12	Ages 13–15	Ages 10–12	Ages 13–15	Total
Low (0–1 events)	1	2	1	1	5
Medium (2**–**3 events)	2	3	2	4	11
High (4+ events)	4	4	3	3	14
Total	7	9	6	8	30

### Exposures to violence and stress

Almost half (46.7 %) of the 30 youth participants experienced 4 or more traumatic events, 36.7 % reported 2–3 and 16.7 % reported 1 or no traumatic events in their lifetime. Exposures to different forms of violence included witnessing the death of a friend or family member, being in a combat situation and forced separation from family and lack of basic needs, such as lack of medical care, lack of shelter, and lack of food were reported among youth participants. The qualitative analysis revealed that exposures to traumatic stressors occur at the individual, family and community level.

#### Individual exposure to violence

The majority of young adolescents interviewed had a personal story of loss and suffering. Participants described experiences of militia groups coming through their village. For example, a fifteen year old girl recollects her experiences with conflict-related violence,

“In the past (2004), we were not sleeping in our houses. Our village was all the time attacked by FDRL (armed combatants) These soldiers terrorized, killed, and raped people, and plundered houses, if I had power, I would kick them out of my country. These crooks came very often, during the day or at night, and inflicted to people horrendous things. They came to our house, when I was 7, and grabbed our property, goats, clothes and other things”.

Youth participants describe armed groups intentions as primarily to plunder or steal items of value from homes. The memory of armed conflict is long lasting and difficult for many youth to forget. These events cause continued anxiety and fear that can be detrimental to mental health. For example, a fourteen year old male recounts.

“What has already frightened me is the thought that the people who come here to kill others can also kill me as I am alone in the house. I am unable to help myself and stop this fear, and so I wish we had people to protect our village. Bandits have already killed a neighbor; I’ve been afraid since then”.

The effects of armed conflict continue to influence youth lives. A ten year old female explains.

“I’m afraid, when I hear gunshots and when people fight in the village, even though I may not know those people. I’m also afraid when I see blood, especially when mothers are nursing people who got wounded in fights. Usually such events upset me, and I usually hide in order not to see what’s going on. One doesn’t forget easily after they saw blood. Sometimes I even vomit”.

Experiencing violence at the individual level may directly affect mental health and well-being outcomes or may be mediated by coping strategies.

#### Violence in the family

*A* common theme from nearly half of youth interviews was the experience of domestic violence, both witnessing violence between parents and experiences of being beaten by a family member. This is an important finding because attachment relationships with parents/caregivers are critical for helping youth to cope with trauma and stress [14]. Family members can act as a protective factor or parents can limit youth coping if they themselves are unable to cope with their trauma [39]. A 12 year old female speaks about witnessing violence between her parents.

*“*When my parents are angry, they quarrel, and dad dismisses mum from the house. Sometimes they fight up to the point of wounding each other. When mum is angry, she barks at everybody in the house. Their reactions are not good: when they fight and wound each other, they have to be taken to hospital for treatment, and pay the money they’d spend on our school fees and food”.

Youth are aware of the significant negative impact domestic violence has on the entire family’s health and economic stability. As explained above, the money used to cover medical costs related to the violence could have been spent on school fees for children or other family needs.

Other youth described triggers of violent episodes in the house. Many youth, pointed to alcohol and it’s role in provoking violent episodes. A fifteen year old male explains.

“When my dad is angry or sad, he beats children, refuses to eat and goes to drink. When he comes back drunk and finds food on the table, he spills it on the ground, and then goes to sleep....Dad’s reaction is not good, because he can wound one of us, and then he’ll need money to rush the victim to hospital”.

A twelve year old girl explained that violence in her family has become more frequent and effects family relationships.

“In past, my parents didn’t quarrel. It’s only these days that they’re quarreling, and my father is more and more absent. When dad comes back drunk, he disturbs the family and fights with mum. I don’t like to see him drunk. In such moments, mum is sad, but after some time she cools down, and things get back to normal. But dad will go away with his friends, and come back late and angry. I hate seeing people barking at each other in the family. It’s good neither for parents nor for children”.

These exemplars indicate that youth witness and experience diverse types of violence in their home, and that they have linked alcohol use and loss of financial resources to the violence in their home.

#### Community violence and threats of instability

Instability in the community and communication between people about the threat of violence can impact youth mental health. Youth participants repeatedly described two threats of violence in the community; La Kabanga and sorcerers (witches) indicating persistent fear and worry by youth. La Kabanga, an in vivo term used by youth and refers to a weapon (a rope used to strangle people) and is also used to refer to people who kill others with this weapon (‘Kabanga people’). Many children described deep fear of Kabanga people. This fear is strengthened by discussion about La Kabanga between peers and families in the community. A ten year old female responded, *“*I’m afraid of the ‘Kabanga’ people (they strangle people with cords). When I heard a child was killed by them in Walungu, I was afraid. I’m afraid of walking alone at night”. An eleven year old male distinguishes between armed combatants from armed conflict and Kabanga people, suggesting that Kabanga people are bandits that kill for no reason rather than being motivated to fight for a particular militia group exclaiming, “I’m also afraid of these bandits who pitilessly kill people at night. I’ve never met them, but people are strangled by ‘Kabanga’ men”. While these threats of Kabanga men may or may not be fictional, belief in the concept of stranglers in the night may cause increased fear and isolation of youth. For example, a fourteen year old male described isolating himself from the community to remain safe.

“When I heard stranglers were killing people in other villages, I was so scared that I couldn’t take a walk in my village, because I thought I could bump into them. In order not to be caught by them, I stayed home, and that’s what helped me survive. But up to now I’m still afraid of them”.

Another threat of violence in youth interviews was violence via “poisoning” from sorcerers, or witches. Traditional beliefs in these settings include belief in sorcerers who may be hidden in the community and who use witchcraft to seek advantage, revenge or to destabilize relationships in families and communities. One 12 year old female recounts, “One day, girls in our village called me a thief and a sorcerer, and said my mother was a sorcerer and a poisoner”. Such accusations have the potential to destabilize community relationships and perpetuate revenge related violence. For example, a fourteen year old male recounts, “I’ve already been angry, especially the day my mother was killed. She had a friend who poisoned her, and then fled to Bukavu. When I consider that I’m motherless, I always say that if anyone shows me my mother’s killer, I can also kill her”. The existence of belief systems around sorcerers and stranglers can perpetuate fear and feelings of community instability. For example, a 14 year old female remarks, “There’s no security in our village because of sorcerers and Kabanga”. These beliefs can be further perpetuated in the community and among youth when there is an unexplained death in the family or community that is attributed to sorcery. For example, a fourteen year old girl recounts, “When my friend died, I was afraid, because she was not sick. She was taken to the prayer-room, and then she passed on suddenly. I felt very bad, because I heard my friend was killed by a witch”.

### Emergent themes: cognitive and behavioral coping strategies

Youth described a wide range of coping strategies in response to experiences of trauma and violence and these strategies were grouped into two domains, cognitive and behavioral coping strategies. The most common cognitive strategies included trying to forget the traumatic event and use of prayer and behavioral strategies included risk taking behaviors and seeking social support. While trying to forget and praying were grouped as cognitive strategies and risk taking behaviors and social support seeking were grouped as behavioral strategies, there existed considerable overlap between these two domains. For example, engaging in play or spending time with friends was reported as a way to help youth “forget”, but it also implies use of social support, particularly if the play is with friends or distraction activity involves spending time with others. The authors grouped distraction activities such as playing with friends under the “trying to forget” theme if the youth stated that the goal of the activity was to help in *trying to forget*. Therefore, the authors caution that while these domains were best supported by youth reports of coping, there exists potential for these domains to be correlated with one another and in some cases would not exist in isolation from another coping strategy. Furthermore, while these strategies have been included in existing coping scales their meaning within the Congolese context may be different. In particular, trying to forget and prayer may be particularly helpful coping strategies, especially in the short term, as youth navigate adaptation trajectories over time.

#### Cognitive strategies: trying to forget

The *most common coping behavior described was “trying to forget*”. Although youth engaged multiple coping strategies to deal with stress and trauma, trying to forget was often described as the ultimate goal in dealing with stress and trauma. Many different activities were described as helping children to “forget it all”. For example, a 12 year old female responded.

“To forget it all, I play with my friends. A little time after I’ve played, sadness goes down. I also share with my friends. We chat about good things that make us laugh, and I feel okay. I also pray or sing in order to feel better. When I’m sad, I do my best to get bad thoughts off my mind”.

Another 12 year old female describes failed attempts to forget despite trying to distract herself.

“One day, I was extremely sad, and I went to a wedding ceremony to see if it’d help me forget, but anger went on burning inside me. Sometimes, I keep myself working (fetching water, for instance), but it doesn’t help”.

Youth described additional ways to forget as coping including prayer, playing with friends, and working. A fifteen year old female suggests that forgetting an event is associated with “moving on”. She describes forgetting an event or feeling as turning the page, “When I’ve taken some sleep or rest, I’m able to turn the page and move on”. Other youth described how seeing ability of others to cope positively might help youth to “forget”. A fourteen year old boy explains, “When I’m sad or angry, I isolate myself. But when I see that other people are fine, I also forget that I was sad or angry. I manage to forget it”. Powerless to change past events, focusing on activities that might lead towards “forgetting” a traumatic event may allow youth to use cognitive distancing to overcome harmful memories.

#### Cognitive strategies: prayer

A second cognitive strategy thematic in the youth interviews was the use of *prayer*. Religion is an important component of Congolese cultural identity. As a support system, religion extends not only to individuals through prayer but also to families and communities by bringing people together in and outside the church.

For youth, prayer was described as powerful for reconciling past events and asking forgiveness, giving strength in the present and providing hope for the future. A fifteen year old girl explains how prayer helps her to cope with past events and forgive and also continues to provide strength in the present.

**“**I’ve been ill at ease, ever since my parents died. I’ve never seen my dad: it seems he passed on, when I was in my mum’s womb. And my mum passed away, when I was 8. My mum told me that dad was killed (poisoned) by someone living in this village. This is no longer a problem to me, because I’ve already forgiven my dad’s killer. When I see this person, I don’t feel any grudge in my heart. When I remember my parents, I only pray – it’s the only thing I can do. Prayer fortifies me, and keeps these sad thoughts off my mind, although it’s difficult sometimes”.

Youth described turning to prayer to overcome maladaptive urges such as seeking revenge. A fourteen year old boy explains that in reaction to his mother’s death he turns to his faith and prays to get rid of urges to seek revenge.

“I can get rid of this kind of thoughts only by praying to God. But sometimes these thoughts persist in my heart even after I have prayed. My father told me that I must pray when I begin to have these thoughts, and that prayer will help me forget them”.

Prayer is a powerful coping behavior because it draws upon a community resource (religious institutions) and connects that resource through individual action (prayer). In this way, prayer is accessible at all times as a coping strategy but is also rooted in and connected to larger family and community support systems.

#### Behavioral strategies: risk taking behavior

Youth also described a range of *risk taking behaviors* that pose physical and psychological risks to healthy development. Youth reported risk taking behaviors including drinking, prostitution, stealing, fighting, seeking revenge, violence and other criminal activity. Girls also reported experiencing pressures to marry early or engage in risk taking behaviors including prostitution. A twelve year old girl described, “Raped girls tend to get married too early because of trauma”. Another important risk taking behavior reported by both boys and girls is use of alcohol. Drinking alcohol is a coping strategy that most youth are exposed to when they see adults drinking. Drinking was viewed as more common among older children than younger children. One twelve year old girl responded about age differences in coping strategies.

“They react differently, because younger children and older children think differently: when younger children are angry, they cry and insult others. But older children can go to sleep, to play, to take alcohol, to smoke, to sing, etc”.

Whether in the family or in the community, alcohol consumption may be a behavior that youth learn to be a “mature” type of coping behavior without fully understanding risks to their physical and psychological development. Youth participants described risk taking youth as “vagabonds” or “street kids”. This description may imply that youth may be driven towards risk taking behaviors as a coping mechanism when family and social support systems are absent.

#### Behavioral strategies: seeking support

A key coping behavior thematic in the interviews was actively *seeking support. Support seeking was done at multiple levels* – from peers, family and community. The qualitative analysis reveals that support seeking strategy is multifarious and contingent on the particular circumstances in which a youth is placed in a youth’s social ecology. The results of the study affirm previous studies while adding depth and detail to this behavioral strategy in DRC. For some youth peers were a source of support. Youth use peer relationships to talk about their feelings but also to engage in activities such as play, sports and singing in choirs that can help get their mind off the trauma or stressor they have experienced. For example, a fifteen year old female responded.

“The advice this friend of mine gives me influences me positively, and helps me deal with problems. It enables me to stop brooding over diverse sad events I have experienced in my life, and I can forget”.

The most frequent source of support discussed was from immediate family. Youth described the importance of family as a source of counsel or advice when experiencing difficult situations in addition to the families role in providing basic daily needs such as food and paying school fees. For example, a fifteen year old female responded, “When I have a problem, I talk to my parents. They’re the ones who understand me easily and who can help me. When I need something, they give it to me. And they give me advice”. Families are an important source of stability and guidance for youth. Youth unable to access family networks for support, particularly financial support, may still seek support as a coping strategy by asking friends and other communities members for help, including work to support financial needs. A fourteen year old female states:

“When I have problems, my family no longer helps me. Nobody helps me, so I’m on my own. If I’m sick, I’ll look for medicine alone. I can ask my friends for money or I can go to a brick-making factory to work for money”.

When family support systems are unavailable, youth may rely more heavily on community support systems to supply the resources typically provided by families. Community members can help support caregivers, helping to guide parenting decisions and ways to support youth through difficult situations. By providing support to caregivers, community members can help to address youth’s needs and work as a unified team to guide youth towards positive coping behaviors. Youth also recognize the importance of community cohesion. For example, one fourteen year old female describes community members looking out for one another, “When I hear gunshots or that there are thieves in our neighbors’ houses, I am scared… Sometimes when we hear that they’re harassing our neighbors, we cry for help. They’ll (criminals) then get scared, and take to their heels”. Community support systems can take a variety of forms. Communities that are cohesive can offer protection to one another in times of need. Communities can offer support to youth through counsel and mentorship such as types of relationships formed at school and church.

## Discussion

Exposure to trauma at the individual, family and community level necessitates that youth employ different cognitive and behavioral coping strategies. Past research has divided coping strategies into two domains, disengagement or emotion focused strategies on the one hand (trying to forget, isolation, substance use) and engagement or problem focused strategies on the other (seeking social support, problem solving, political participation) [25]. This perspective tends to assert that disengagement/emotion focused strategies are negatively associated with mental health while engagement/problem-focused strategies are positively associated with mental health. The current study along with more recent research has sought to complicate this normative perspective on coping strategy to better understand and redefine coping strategies within specific cultural contexts. Furthermore, this study highlights the need to understand potential relationships between coping strategies and ways that cognitive and behavioral strategies can be mutually reinforcing with the potential to help or harm youth well-being.

In eastern Democratic Republic of Congo, different types of cognitive and behavioral coping strategies may be tied to the post-conflict and sociocultural context for youth participants in this study, thus underscoring the importance of context in understanding coping strategies. Where youth may have been limited in their ability to engage with the traumatic event directly (a kind of “problem solving” strategy), youth may turn to alternative strategies such as “trying to forget” and praying. These cognitive strategies have been described as “disengagement strategies”, however it is unclear whether in this context these strategies should be considered to negatively affect mental health.

For example, a qualitative study on coping in Sri Lanka after a tsunami disaster found that many participants found that keeping busy and distracting oneself could be a successful way of dealing with stress [40]. The research found that, “many engaged in work and leisure activities and religious rituals as a way of providing relief from their troubles” and found that these activities fulfilled a dual purpose of meeting practical needs (income and livelihood generation) and psychological and emotional distraction [40]. The participants in this study found that these types of distraction activities were especially important in the immediate aftermath of the disaster and were described as an engagement strategy in early stages of recovery.

Trying to forget may also be representative of a kind of cognitive flexibility, which refers to the ability to “reappraise one’s perception and experience of a traumatic situation instead of being rigid in one’s perception” [41]. Cognitive flexibility allows acceptance and assimilation of a traumatic experience into one’s life and can provide opportunities for growth and recovery. Prayer and faith, a common coping strategy utilized by participants, may be a coping strategy that works as a form of cognitive optimism. Optimism has been conceptualized as the maintenance of positive expectations or hope for the future [42]. Research argues that cognitive flexibility, together with optimism can allow an individual to demonstrate resilience while accepting their current reality [41].

This study found that family and community support can be protective to youth or can act as a risk factor for negative outcomes. As a protective factor, families provide basic needs, provide safety and security and are a source of material and psychological support. A study in northern Uganda among 741 former male child soldiers found the role of the family was critical to long term mental health outcomes [43]. Family dynamics between mother and father are also important. A qualitative study among 86 Palestinian youth affected by conflict, found youth who perceived mothers as loving but not fathers had higher levels of PTSD symptoms as compared to those with parents they both considered loving [44]. As a risk factor, domestic violence can be an ongoing stressor for youth and can result in inability to meet basic needs including food, school fees and health care. The multiple trauma and stressors likely have a cumulative effect and may also impact the types of behavioral and cognitive coping strategies used by youth. As a mediator coping strategies may be partial (account for only part of the effect from traumatic event (s) to outcome) or total (account for all of the effect from traumatic event (s) to outcome). Considering trauma exposure on the individual, family and community level adds complexity to the ways we consider trajectories of resilience.

Given the collectivist nature of Congolese identity, community relationships have a role in shaping coping strategies. In this study participants sought support among peers, siblings, family, teachers, churches and other community members. Research has found that social support and feeling connected to neighborhoods and schools is associated with better mental health outcomes in children [45] Prayer and religious faith was a common coping strategy utilized participants and connect individuals with religious support systems. This can be a key coping resource for individuals, particularly where they feel able to ask questions and gain council about their traumatic experiences. Faith may also work to provide people with connection to religious support systems where people feel free to ask and gain answers about traumatic situations. Research indicates that positive religious coping has a moderate positive association with psychological adjustment [46].

While community support systems are important resources for youth, these systems can also contribute to processes of fear through interpretation of illness as being caused by sorcerers or witches. Intervention approaches aimed at improving coping strategies in youth should collaborate with local leaders to develop approaches that are non-judgmental towards traditional belief systems of causes of illness but rather work in dialogue with leaders to develop interventions that minimize negative effects of these beliefs such as perpetuating fear leading to social isolation. Social support seeking also has the potential to lead to risk taking behaviors. For example, it is plausible that some peer-support seeking could potentially increase the likelihood of engaging in risk-taking behaviors such as drinking, a negative coping behavior usually employed in social settings. Supporting positive group activities for youth could extract the benefits received through socializing in peer networks and could potentially deter youth from utilizing risk-taking behavior in social groups.

Understanding the complexity of coping among conflict-affected youth in the context of the DRC helps develop a more complete theory of cognitive and behavioral coping strategies that is helpful for modeling pathways for empirical testing. For example, unlike previous research, this study reveals that disengagement strategies can be an effective coping strategy within this context. Reliance on the western constructs of coping may inappropriately prioritize certain coping strategies as beneficial, such as engagement or problem-solving strategies, when these types of strategies may be of secondary concern or simply lacking meaning in contexts where youth are impacted by conflict related traumatic stressors. In addition to recognizing use of a particular strategy, this study identifies the possibility of overlap between coping domains and mutually reinforcing relationships between particular strategies that could potentially help or harm youth well-being. Future research could benefit from a more complex understanding of the relationships between coping strategies and potential reinforcing relationships between cognitive and behavioral strategies. A context specific framework can provide a springboard for implementing effective intervention policies.

### Limitations

Results from this study may not be generalizable to other contexts as coping strategies were defined within the cultural context of the Walungu Territory in Eastern Democratic Republic of Congo. The villages sampled in this study were rural villages and coping strategies in urban contexts may differ if additional resources and support systems specific to urban environments are available. The youth included in this study had a wide range of trauma exposure related to ongoing armed conflict and it is possible that coping strategies change over time post-conflict. Future research and public health programming should consider adaptive trajectories over time.

## Conclusion

This qualitative research provides a culturally specific portrait of coping in the Walungu Territory, Eastern Democratic Republic of Congo. While traditionally coping strategies grouped under the “disengagement” domain have been construed as negative coping strategies, in Eastern Democratic Republic of Congo types of disengagement coping such as *trying to forget* and *praying*, may help to support youth mental health along adaptive trajectories. Coping strategies are related to risk and protective factors at the individual, family and community level. Having attachment relationships with peers, family and community provide stability and structure as well as an opportunity for emotion expression. Greater cohesion and integration of family and community in intervention efforts can better support strength based interventions for youth.

## Abbreviations

DRC: Democratic Republic of Congo
PAIDEK: programme d’Appui aux initiatives economiques
PFP: pigs for peace
RFR: rabbits for resilience
